# Cannabis Induced Psychosis

**DOI:** 10.1192/j.eurpsy.2022.45

**Published:** 2022-09-01

**Authors:** J. Bramness

**Affiliations:** 1 UiT-The Arctic University of Norway, Institute Of Clinical Medicine, Tromsø, Norway; 2 Norwegian Institute of Public Health, Department Of Alcohol, Tobacco And Drugs, Oslo, Norway; 3 National Advisory Unit for Concurrent Substance Abuse and Mental Disorders, Innlandet Hospital, Ottestad, Norway

## Abstract

Even if most people use cannabis without many negative consequences, some experience cannabis related harms. At higher levels of intake, one of these harms could be psychotic symptoms and even cannabis-induced psychosis. During the last years, we have seen increased treatment seeking for cannabis use disorders in Europe. Parallel with this increase we have seen an increase in the incidence of cannabis induced psychosis in all Scandinavian countries and an increased population attributable fraction for cannabis on the prevalence of schizophrenia has been demonstrated. All this may reflect increased use of cannabis, but more likely increased content of D9-tetrahydrocannabinol (THC) is most cannabis products. Many have also pointed to the fact that we have seen an increase in the incidence of schizophrenia in the same period, maybe attributable to cannabis use. If we also take into consideration that up to one third of those with cannabis-induced psychosis over time receive a diagnosis of schizophrenia, we may see at least two implications. Firstly, these findings strengthens the evidence for a causative relationship between cannabis use and schizophrenia, a causative relationship that man have suggested for several years, but that has had some opponents. Secondly, it points to cannabis-induced psychosis should be considered to be a part of the ICD-10 F2-chapter rather than the F1-chapter. This may be true also for the other substance-induced psychosis (F1x.5). An additional argument for this is that for no other psychotic diagnosis, a precipitation factor is mentioned in the diagnosis.

**Disclosure:**

No significant relationships.

